# Being a woman researcher in an Anatolian village

**DOI:** 10.1186/1746-4269-9-45

**Published:** 2013-07-02

**Authors:** Füsun Ertuğ

**Affiliations:** 1 Orhangazi Cad., Kumbasi Yolu no: 109, İznik/Bursa 16680, Turkey

## Abstract

This essay represents the first editorial of the series "Recollections, Reflections, and Revelations: Ethnobiologists and their First Time in the Field". In this memoir, the author details the evolvement and intellectual progression of her research focusing on wild food plant consumption within a remote community in the high steppes of Central Anatolia during the early Nineties. The author conveys a human learning journey as a woman and an ethnobiologist, reflecting on the methodological bottlenecks and solutions during her first ethnographic experience in the field.

## 

I was luckier than most anthropologists and ethnobiologists as ‘my field’ was not in a foreign country. I had known my fieldwork area for at least three years when I started to research it. The language of the people of the area was my mother tongue. I shared the same religion and culture. I went to the village as a member of an archaeology team that had been excavating a nearby mound called Aşıklı^a^ since 1989. My first visit to Kızılkaya village was in 1990, and I selected this area as the topic of my Ph.D.^b^ thesis [1] in 1993, and went to settle there in January 1994. However this time my situation was different than when I was a summer guest, so I started to make friends as a village resident-guest. It was my first long-term study, but also I was the first researcher to go to settle in this Central Anatolian village.

‘My village’ was located on the high plateau of Central Anatolia, and was 25 km southeast of the town of Aksaray. It had a population of 1300 (over 300 houses) Sunni Muslim Turks and according to historical documents the village of Kızılkaya had been there for at least 500 years. Its economy was based on agriculture, basically field cropping and gardening, as well as sheep and cattle husbandry. The area is within the Irano-Turanian floristic region dominated by treeless steppe vegetation.

The aim of my thesis was gathering information about the local subsistence economy of the present and the recent past, in order to draw more accurate conclusions about the subsistence of the Neolithic inhabitants of the same area. My work started as an ethnoarchaeology thesis and somehow ended up as a mixture of ethnobotany and ethnozoology in addition to ethnoarchaeology.

My home was in Istanbul, while the village I was going to live in for about 18 months was in the province of Aksaray, about 700 km to the east. Originally I intended to settle there in the fall, after my son’s school opened in September, but as my scholarship checks came quite late, I went to the village in mid- winter. I drove there in a second hand Russian Lada Niva jeep that I had purchased recently with my grant. My jeep was full of books, notebooks, plant presses, camera bags, clothes, boots, a blanket, a rug and so on. I had rented a one-room house in the old section of the village before I went. This section had been partly abandoned in the 1970’s due to stone falling from the cliff behind the old village (Figure 1). The government helped the villagers build new houses in the eastern part, and only a few old couples and poorer families continued to live in the old section.

**Figure 1:**
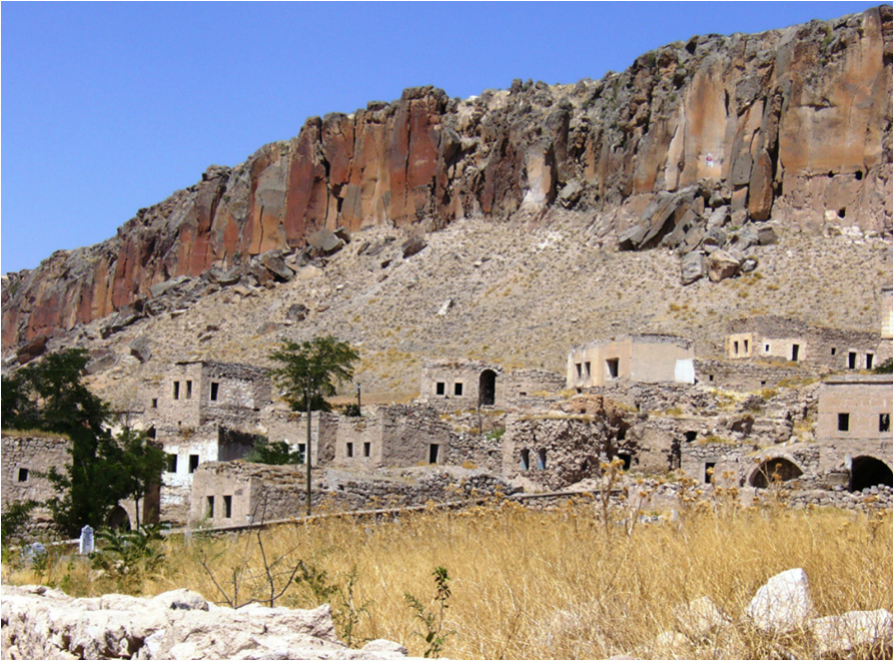
Old section of Kızılkaya village under the cliff, most houses were abondoned in 2006.

Although it was mid-winter when I arrived, there was no snow and I settled in easily with lots of help from the neighbours. It was an old stone house with a single window that framed a beautiful view of Hasan Dağ, a volcano over 3000 meters high that dominated the whole plateau and the Melendiz River (Figure 2). Its wooden door opened onto a small patio where I could see the imposing cliff behind my house. My patio was actually the flat beaten-earth roof of the abandoned barn below, where I kept wood. The house had electricity but no running water. The toilet was outdoors, a circular stone building without a door or running water, with a nice view of the cliff and an abandoned house. I could fetch water from the fountain or from nextdoor neighbour, carrying it in plastic containers.

**Figure 2:**
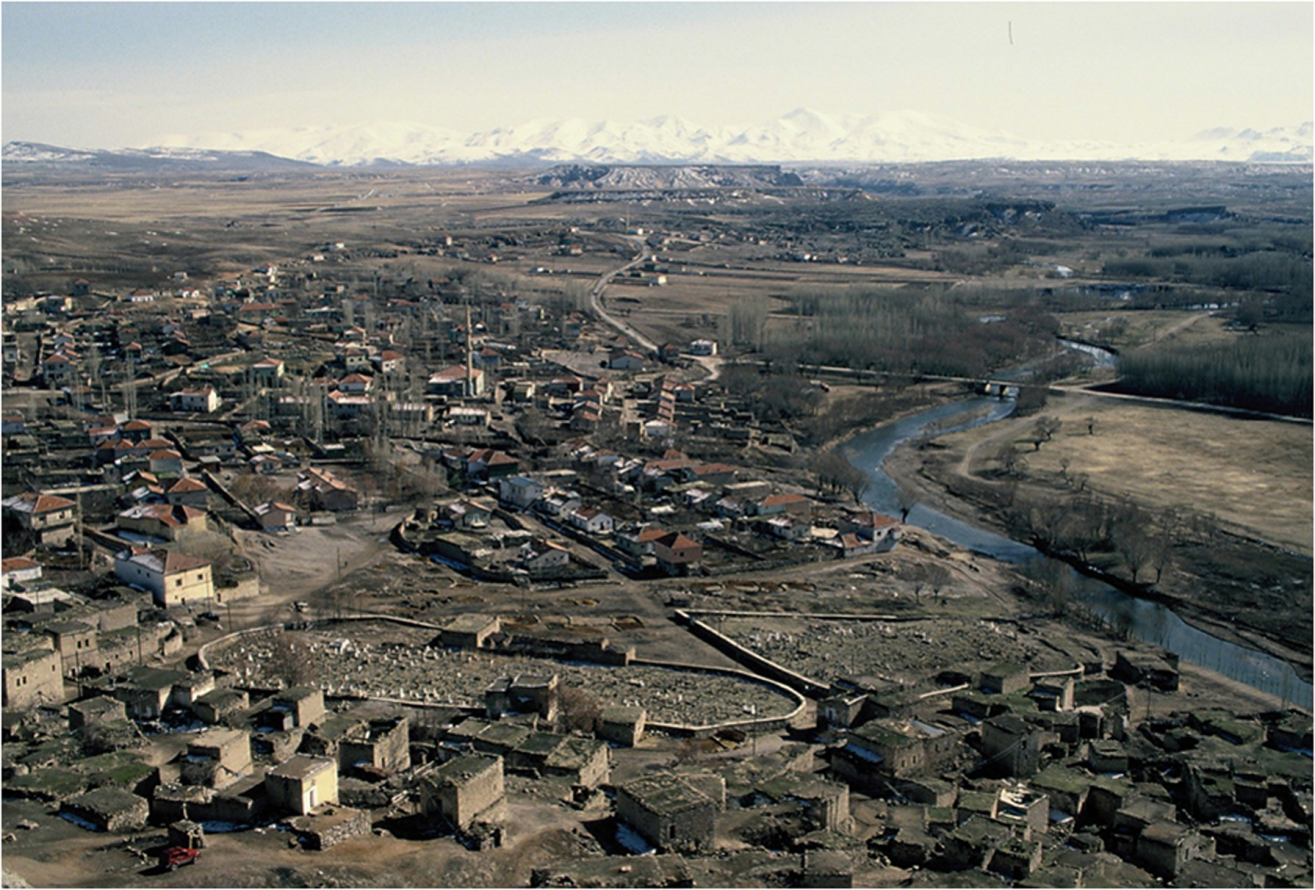
**A general view of the Kızılkaya Village and the Melendiz River from the cliff.** The Hasan Dag volcano and Melendiz Mountain ranges are under the snow on the horizon. Bottom part is covering the old section of the village, while my red Lada Niva is on the left hand corner, parked behind my room. Asikli mound is taking place at the circle of the river behind the bridge, partly hidden by the poplar trees.

I discovered that my room already contained a single bedstead and a wood stove and the walls had been whitewashed by our excavation site guard, Naci Kayan. The room also had a rectangular cement basin, connected to an outside drainpipe, and here I could wash myself as well as my dishes. For a day or two I stayed in the village headman Ismail’s house at nights, while preparing my room. Within a few days I had gathered an old school desk, a mattress and a few pillows from a neighbour, a carpet and a quilt given by someone I knew in Aksaray, and I bought a mat, a small gas stove to cook on and some kitchen utensils. When the village carpenter had helped me to fix the cracks in the old wooden door and built a few shelves, my 4 × 5 m room was ready to accommodate my guests and myself. My neighbour Cennet helped me to clean the initial dust in my room, and I borrowed her vacuum cleaner whenever I needed it. When I tried to wash the wooden beams and reed mat on the ceiling, which had turned black by the soot of years, Cennet also ran to help.

Cennet was my next-door neighbour, a young woman who had come as a bride from Göstük, the village closest to Kızılkaya. She was the mother of two children, Sema (4) and Ramazan (2.5) (Figure 3). Her husband was a construction worker (painter) in the town, but was at home for the winter. Usually young married women with small children live together with the parents of the man, but in their case they had quarrelled with his parents and settled in a two-room rented house. From the first day Cennet became a friend without any conscious effort. She was clever, easygoing and talented. She cooked tasty food, did nice embroideries, and took care of her children very well.

**Figure 3:**
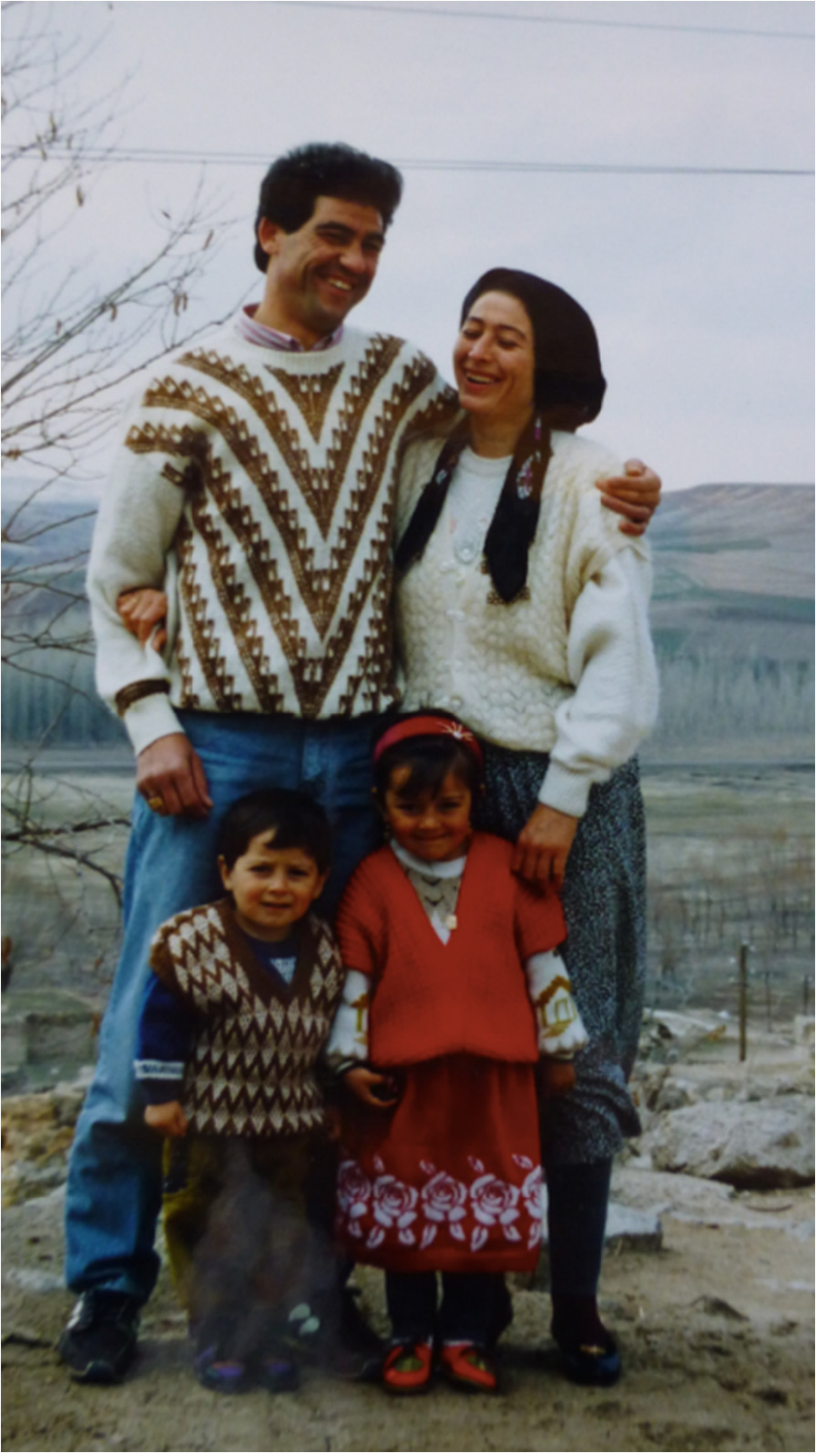
Cennet and her husband, with their children Ramazan and Sema.

From the first night on my small room became an attraction point, a kind of meeting room, especially at nights. A few of the closest neighbours came first, and then the circle was extended. An elderly couple, Mustafa Sagdıç and his wife Fadime, whose house was just opposite mine, came to welcome me on the morning of my third day. Fadime Ana, "mother Fadime", brought four apples in a plastic bag. Usually the women sent their children at sunset to ask if I was free that night, then they would pay a welcome visit with their children. They brought bundles full of *yufka*, local flat bread, gathered greens, an apple or an orange, a cup of soup, milk or yogurt. Once I found a sack of onions at my door, left by someone who had heard that I was asking for onions at the local market. Nobody sold either onions or garlic in the village market, as every family grew their own.

I was very enthusiastic about these evening visits, as my guests not only brought food but many new concepts and information too. I could not forget my excitement when I opened the bundle of gathered greens brought by Tülay (Figure 4). It was my first night in my new home. She told me that they had gathered them during the day and I might like to taste them. She showed me how they eat greens by selecting a few of each variety, placing them on a flat bread, adding a pinch of salt and rolling the bread up. I tasted first the freshness of them, and then the bitter taste of *Chichorium* mixed with *Nasturtium*’s softness. After I finished my roll of greens I tried each species separately, trying to remember the local names. I did not want to lose any of this valuable information, so I was taking notes surrounded by at least five women and four giggling children. I had to postpone my enthusiasm for getting more information, since as the host I had to prepare and serve tea to my guests. The next morning I was at Tülay’s door, a house on the street below my house. I photographed the greens in her bundle, took press samples, asked and wrote down their names and extracted a promise to go gathering together.

**Figure 4:**
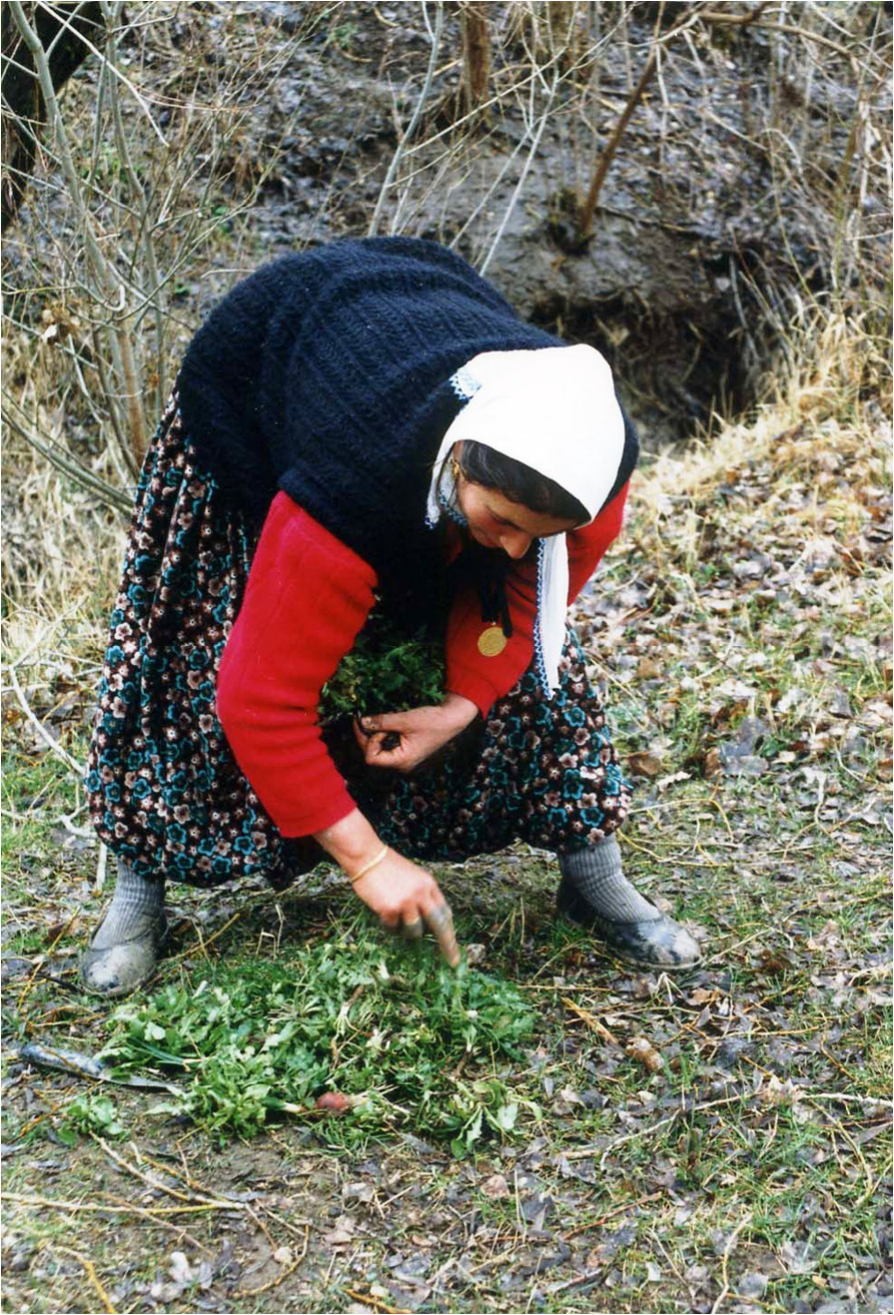
Tülay with her apron full with gathered greens.

While I was writing the proposal for my thesis I was hoping to find a few elderly people who might remember which plants were used for what. As the gathering and knowledge about plants is the feature most analogous with the Aşıklı population who lived there about 9000 years ago, this information was very important for me. However, almost no literature was available about the ongoing tradition of plant gathering in Anatolia. There were some studies about medicinal plants, but they were not the results of fieldwork in a particular area. There were surveys of medicinal plant use, but no one knew which and how many medicinal plants were used in any one village. We had had no idea if traditional knowledge about plants in Anatolia was alive or not. And there was very limited information about edibles. A book compiled by a foreign woman who lived in Bodrum in the 1970’s was the only book on the subject, and she told us that the tradition of consuming wild gathered greens was limited to coastal areas [2]. Yet I was witnessing the tradition of plant gathering by young women in the middle of Anatolia, and tasting various species of greens that my neighbours gathered. This was more than I could hope for. From the next day on I went on gathering trips first with Tülay and her daughter, then my friend Besime and her daughter Ayse, and so on (Figure 5). That was the beginning of my ongoing fascination with ethnobotany. The information gathered in Kızılkaya about wild plant gathering [3-10] also opened up a new era for a generation of young ethnobotanists.

**Figure 5:**
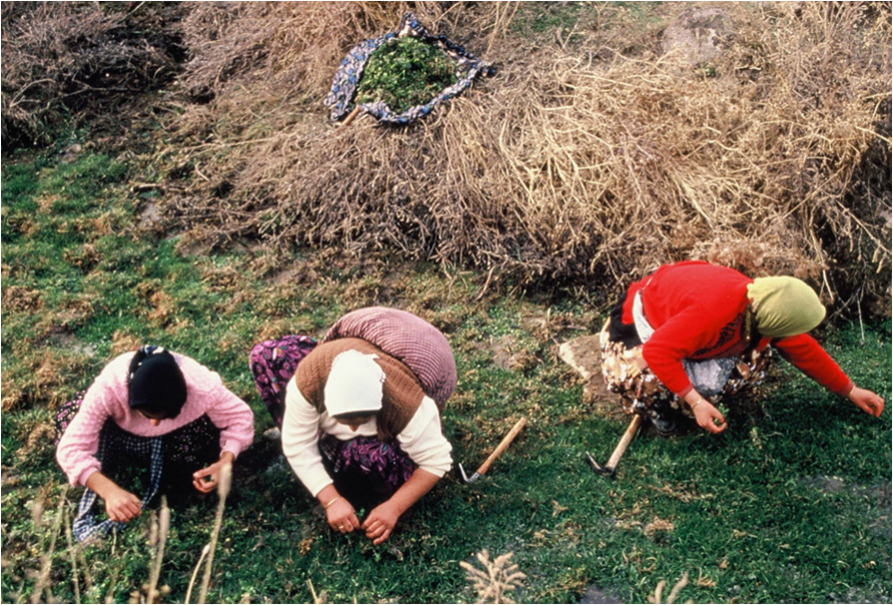
**A group of women in a gathering trip of greens.** See their adzes and a bundle of green at the background, and another one over the back of the woman in the middle.

Besime was already a tutor and friend from my previous visits (Figure 6). She was our excavation guard Naci Kayan’s wife, an imposing mother-goddess-like woman and the mother of three. We discovered that we were born in the same year and always felt very close. She was very clever and wise, and often regretted that she could not even finish elementary school. She had to become a mother to her younger sister and brother when she was still a child, and so had no opportunity to finish her education. I always explained to her what I was planning to do, and went ahead only after we had discussed it. No plan would go right without her consent and review. She knew well who was knowledgeable about what, and often arranged a visit to the person together to introduce me. She was an expert not only about plants, but human relationships within the village. The only times you needed to avoid her were the days before it was her turn to bake bread, particularly winter bread. She would then be very tired of preparing the dough, cleaning the tandur area, preparing the wood, and finding helpers, so you needed to beware of her temper. Except for those few days she was a great tutor, always available, ready to share her knowledge, happy that she was so useful in my research. I was fed by her and taken care of when I was sick, bored or low-spirited.

**Figure 6:**
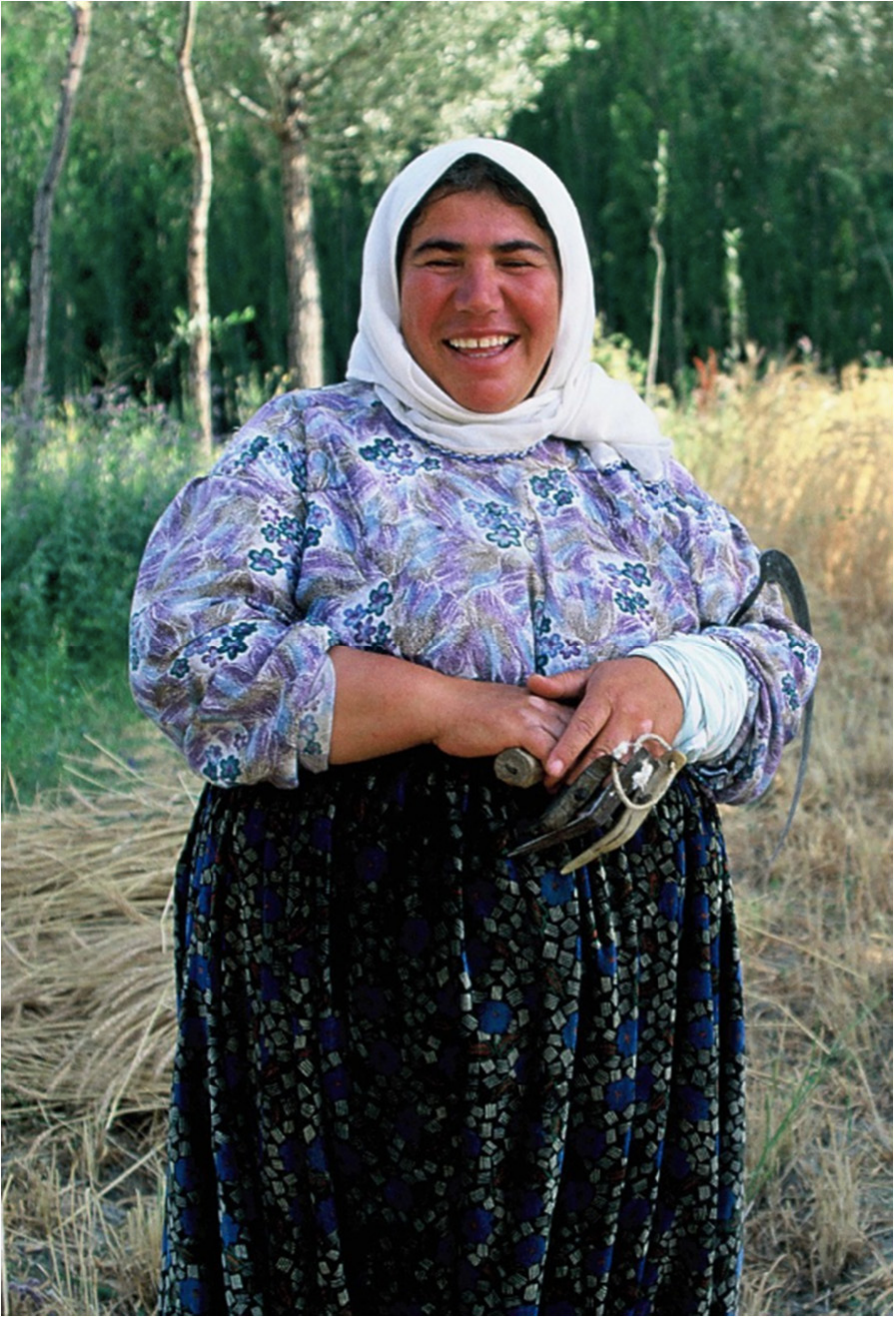
Besime Kayan, my peer, tutor and friend in Kızılkaya.

Besides Besime, I used to visit a few old ladies when I had a headache or felt too low-spirited to continue asking questions. I used to call them *ebe*, meaning grandma, a common term of address for elderly women. One of them was my neighbour, Ümmühanı Ebe, who always brought either a cup of milk or rice pudding when visiting me (Figure 7). She was tall, had a very peaceful face, spoke calmly and held herself proudly. Once she told me her story and we both cried. She was the daughter of a well known and rich man, married her first husband when she was 20, and gave birth to a daughter. Then her husband was away doing his military service for 3–4 years. Another man, her late husband was interested in her and asked her to run away, even though he was also married. She told me she did not know how she ever accepted, but she ran away with him. Her family, her father, mother and brothers were offended, her husband’s family did not let her see her daughter, and nobody talked to her. At first she lived with her new husband’s first wife in the same house, then the co-wife left and returned to her father’s home. When her first husband returned from the army, she and her new husband went to jail for some months. While her second husband was doing his military service her father-in-law tortured her. She told me they had three grown up daughters and four sons, two of whom died in infancy. Her first husband also remarried, but always loved her, and after all those years she still regretted leaving him. After my fieldwork was over, every time I visited the village I visited her house and we sat together, without talking much, but holding hands. She died a few years ago, and I felt as if I had lost a second mother.

**Figure 7:**
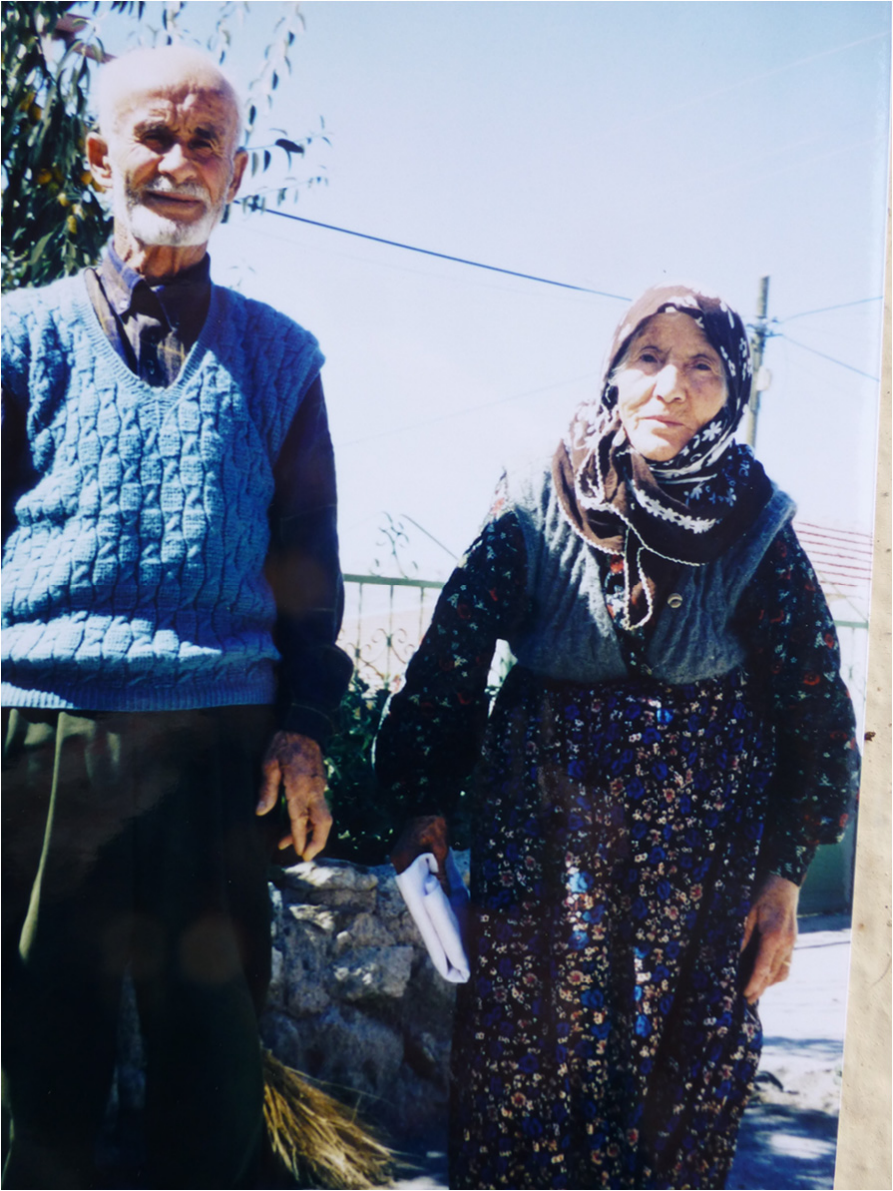
‘Ümmühanı ebe’ with her husband Ali in front of their house in 2002. We lost them both in the past few years.

For about a month, almost every night I was busy trying to keep the stove alight by adding wood once in a while, and serving fruit, biscuits and tea to guests in my room without enough glasses, spoons or plates. At the same time I was trying to gather information about this subject and that, completely unsystematically. Still, it was valuable experience, as I started to learn who had good relations with whom, and who was well informed about what. On the other hand it was a hard task remembering all the names, explaining why I was there, and answering inquiries about myself to the same or different guests every night.

‘I am from Istanbul. Yes, my mother is alive but my father is dead. Yes, I have only one child, no my husband is not against my going to work and being alone in a village. He is an architect, living in Istanbul, and too busy to come and visit me.’

Actually I was divorced when I went to the village, but apart a few people that I knew well in the village, I decided not to tell anyone that I was a single woman. In addition to that I was a mother, who had left her teenage son unattended in Istanbul, the city of sin and crime. Although I was about 40 when I was living there I had been told that some people might assume I was a free woman and harass me, and some others might think that a divorced woman was not respectable. This was nonsense, and today I believe I could have been open about my situation and still no one would have harassed me or thought I was not respectable. However, whenever I was in a village or talking with someone from a village I realised that the concept of ‘privacy’ was different for urban and country people, and in fact that there was not even a word to express this concept exactly in our language.

I have had a few experiences of ‘protection’ by villagers. On one occasion a British botanist friend visited me and wanted to see my herbarium specimens. I invited him to my room, but thinking that it might be misunderstood by the neighbours, I left the door open. It was a summer day, so there was no problem. While we were looking at plants and discussing them, one of my neighbours entered the room and started to call her son from my window. When I asked about it she said that she could not see him, and was worried. Actually my outdoor patio would have been a better place to call her son, but she wanted to show that I was under her supervision. The men of the village also protected me, and asked where I am heading and for how long, each time I left the village by car. When I asked a woman to come with me by car to show me a plant or go to a wedding in another village, we used to ask permission of her husband or father. Usually women liked to come with me, as such occasions were rare.

After a month, welcome visits ended but sporadic visits to see how I was or bring me food or something I had asked for continued. Meanwhile I was visiting as many houses as possible, trying to talk with everyone. Soon I learned not to trust my first impression about a person’s knowledge but neither to dismiss anyone. She or he could have a sour face and look dull or not know what you were asking about, but might know other things that you never thought to ask. On one of these occasions a woman with a very sour face among a friendly group of women told me that she craved for a type of clay and went to dig it up and eat it. When I started to ask others, I found out that geophagy was a common practice among women, probably caused by a need for additional amounts of certain minerals. There was a mentally handicapped young man named Attila who worked as a labourer on the excavation site. He used to call me *hoca*, meaning teacher, and always asked when we were going to dig there again. He also visited my room with two of his cousins, and while drinking his tea told me that I had the most beautiful house in the village. When someone was looking for me, he became his or her guide and found me wherever I was, and was always helping me to carry something or find someone.

After a few months I was able to accumulate lots of notes about so many different things that I felt the yarn that I was spinning was growing longer. I had watched people spinning yarn, and always been astonished how all those short fibres of wool stick to each other to become an even thread. I did not know at that point if my yarn was sufficiently even or long enough to weave a rug. Nor was I sure what colours I needed to weave it. Still I was leaving the house every morning with a rough plan of whom I was going to talk to, and about what. Making appointments did not work in the village. Everything was liable to change and you had to adapt yourself to each new situation, so I did my best.

After about 10 months, my next-door neighbour Cennet moved away and the electricity of my house was cut off without any warning. Then we figured out that my landlord had refused to pay earlier bills and so the local municipality had cut off my electricity. I either had to pay a huge sum or move somewhere else. I chose the second alternative, after paying for the electricity I had used. Someone suggested another one-room stone house in the newer part of the village. I moved there in mid-October, and spent the rest of my research period in this house, which did not have a view of Hasan Dağ, like my previous home. The house was in a courtyard, and I could see apple trees from my window. It had traditional stone arches and high ceiling but no running water or indoor toilet. There was a faucet and a toilet that was better than the previous one, possessing such features as a door, in the courtyard. A somewhat similar ceremony of welcoming visits occurred in the new quarter, but lesser in number. Everyone used to see me everyday in the village.

I had a three-day long mouse hunt in this new house. It was at the end of November, the weather was cold, and one night I heard a rattle. When I turned on the light, I made eye contact with a small mouse sitting over the plate shelf. It ran away and I ran after her with broom in hand until dawn. The next day I found her inside my suitcase under my bed, and she ran away again. I asked Besime’s daughter Ayse to help, but she refused. However, Naci came to help and we carried everything outside. Although it was snowing we emptied the house and poured hot water to the corner basin where I used to bathe, thinking that the mouse might hide in the pipe. All in vain, however, because the next night she was again watching me and running over my books. This time I attacked her with a roll of maps. Many people suggested a variety of ways to get rid of the mouse, including keeping a cat, and putting down traps or poison, but no one offered to help. Meanwhile I heard all kind of funny mouse hunting stories. It was clear that village women were as scared of mice as much as I was, or even more. Next I approached the elementary school teacher Dilber, who had told us about catching eight mice with a broom in a single night. With her help we finally get rid of the mouse and closed up all possible cracks, pipe holes, etc., but washing all the plates, glasses and pans, and resettling in the house in snowy weather was much more difficult. A tiny mouse caused three sleepless nights, and attacking her like Don Quixote had exhausted me to bone and created anxiety for about a week. For some time everyone in the village kept asking me if I had any more mice at home, smiling hugely as they did so. Evidently many people found the incident amusing.

As a researcher and a guest in the village I had to be polite and smiling all the time. So I tried my best, and most of the time at least I was happy and smiling. If I was not, everyone heard immediately and asked if I was sick or whether anything was wrong. One Sunday morning I was squatting in front of my door, trying to wash my clothes in a plastic basin with warm water heated on the stove, and answering the greetings of passers-by. I told them that I was washing my clothes, yes, everything was all right, and no, I did not have any troubles. After I don't know how many repetitions, I told someone the same thing with a trace of impatience. The same afternoon, when I was at the other end of the village, people asked me if everything was all right, as they had heard that I was a bit annoyed that morning.

I had prepared many questions about gathering, agriculture, including field size, crop type, decisions about which crops to plant, animal husbandry, herd size, calendar, etc. The main problem was whom to interview and how to ask all these questions in a systematic way. The question of ‘whom’ was solved with the help of many people. For animal husbandry I had a few good informants, and for agriculture many people volunteered. Gathering edible greens and information about medicinal plants and applications were discussed either among small groups of gatherers or with groups of women when they visited a house for celebratory or social occasions such as celebrating a child’s new tooth, or winter night gatherings. Actually the best way of learning was doing it, and by attending every activity, from harvesting to planting, from gathering to processing, I used to learn and share the difficulty as much as the joy.

When a list of edible plants started to accumulate, I began showing and reading my list, and the women would remind me of another name or approve my list. The women who gathered all these greens were excited to see the increasing numbers. When a new one appeared on the list everyone asked who had made the addition. A few plants were not known by everyone, because the informants were brides from other villages, and the ones they gathered were not known or preferred by Kızılkaya women. The solution was to add a star to those that were not accepted by everyone. The children were also very helpful; particularly boys, who had a habit of going out to collect mushrooms and *Crocus* like bulbs. They liked me to take their pictures with their knives and mushrooms or bulbs. Their mothers also appreciated their contribution to the family table.

Information about medicinal plants was not as evenly spread as for edibles. Some women were more knowledgeable than others. There were also specialist healers, only one of whom used plants, while others healed by means of prayer or other methods. We visited some of these healers with the women of Kızılkaya. When someone’s child got sick they used to ask me to drive them to the healer's house, and by this means I observed actual healing processes, instead of just obtaining descriptions. Besides these specialists, some women had healing powers or knowledge of how to relax by touching. I did not include such non-scientific information in my thesis, as these healers were not using any plants, prayers, or any other known techniques. You laid your head in the healer's lap, and she held your head, slowly massaged it, and when they started to yawn, your headache, your pain or anxiety disappeared. Years after, when I was in Southern India for a medicinal plants conference I watched healers similar to these old ladies.

I was collecting and pressing the plants used in the village, but I had no idea about their genus or species, as my knowledge of botany was insufficient to identify them. I had attended botany and paleoethnobotany courses, and learned how to press plants in St. Louis, but that was all. When my proposal was accepted I received permission to take my plant samples for identification at Missouri Botanical Garden (MoBot). However, no specialists on the plants of Anatolia were available at MoBot, and I did not know any botanists in Turkey. I used to look up their local names in Turkish reference books and try to learn their Latin names. Someone gave me the name of Prof. Tuna Ekim, head of the Botany Department at Gazi University in Ankara. When I had collected about 300 or more samples I finally called him and asked if he could help me. It was the beginning of November 1994 when I packed up my plants in the back of my jeep and drove to Ankara to meet him. He welcomed me and introduced me to his team. Prof. Mecit Vural, who was then curator of the Gazi Herbarium spent many hours with me and sent lists of identified plants so that I could check them in books. So another long-term and very productive relationship started and has continued up to the present day. The Gazi University team not only completed all the identifications, but taught me how to use the herbarium, introduced me to some other specialists for particular genera, and they even came to the village when I needed help. I owe a great debt to Ekim and Vural and all their team members.

After a year, during my second winter in the village, I started to fill out some questionnaires based on interviews with a randomly selected group of 30 families, to be able to have some quantification. Before selecting them, I obtained village tax information from the headman, and used their tax division into three groups: poor, middle-income and wealthy. By selecting 10 families from each group I obtained more or less balanced data from the village. I asked the woman of the household for information about gathering, and the man about agriculture, land size, crops, etc. In a few cases where the man was not in the village, the woman answered both. With respect to animal ownership this type of data was inadequate, as herd composition was important information and who owned what did not work by sampling. So with the help of Mustafa, Besime’s nephew who also owned animals and who knew everyone in the village, we compiled a census for each house. Everyone answered seriously when we asked the number of their cows, sheep, donkeys, etc., but many laughed when we ask about the number of their chickens. An infectious disease had killed almost all the chickens a few weeks previously, so we got answers such as ‘used to be 20, now zero’ or ‘a few left’.

Towards to the end of my research I still had many questions without answers and a few plants that I was not able to gather in time to have them identified. My advisor at Washington University in St. Louis, Patty Jo Watson, came to visit me in the village at the beginning of June 1995, and stayed a week while we discussed the data I had gathered. She told me that the Ph.D. is only a step, and that no one can collect all the data and be able to answer all the questions. It was better to see what I had collected by writing it down, and I accepted her wise suggestion. I needed to see if I had enough yarn to weave a rug.

I stayed in Kızılkaya village from the beginning of January 1994 to the end of June 1995, a full 18 months, except for brief monthly visits home and for seeing specialists. I have returned there almost every year for the last 18 years. I have seen babies grow into children, and attended their weddings. My friendship with some people faded over time, but some women became my lifelong friends, sisters and aunts, and I also have some brothers and uncles. I have shared sad moments, lost some of them, gone to their funerals or visited the bereaved family when I went to the village. As well I have shared happy occasions such as marriages, newborn children and the return of young men from military service. I have learned so many things from them and feel deeply at home when I am in the village. I have studied several other areas since 1995 and become friends with other people; however, I owe my deepest thanks always to the villagers of Kızılkaya who accepted and protected me, shared and provided my first field memories.

## Consent

The persons involved in this manuscript has given their oral consents to the author for the report to be published.

## Endnotes

^a^For more information about Aşıklı mound see: http://www.asiklihoyuk.org/AHeng.html

^b^I was doing my doctorate at Washington University in St. Louis, USA.

## Competing interests

The author declares that she has no competing interests.
